# Order-Stability in Complex Biological, Social, and AI-Systems from Quantum Information Theory

**DOI:** 10.3390/e23030355

**Published:** 2021-03-16

**Authors:** Andrei Khrennikov, Noboru Watanabe

**Affiliations:** 1 International Center for Mathematical Modeling in Physics and Cognitive Sciences, Linnaeus University, SE-351 95 Växjö, Sweden; 2 Department of Information Sciences, Tokyo University of Science, Noda City, Chiba 278-8510, Japan; watanabe@is.noda.tus.ac.jp

**Keywords:** biological, social, and AI systems, order-stability, classical vs. quantum entropy, quantum channel, entanglement, quantum-like models

## Abstract

This paper is our attempt, on the basis of physical theory, to bring more clarification on the question “What is life?” formulated in the well-known book of Schrödinger in 1944. According to Schrödinger, the main distinguishing feature of a biosystem’s functioning is the ability to preserve its order structure or, in mathematical terms, to prevent increasing of entropy. However, Schrödinger’s analysis shows that the classical theory is not able to adequately describe the order-stability in a biosystem. Schrödinger also appealed to the ambiguous notion of negative entropy. We apply quantum theory. As is well-known, behaviour of the quantum von Neumann entropy crucially differs from behaviour of classical entropy. We consider a complex biosystem *S* composed of many subsystems, say proteins, cells, or neural networks in the brain, that is, $S=(S_{i}).$ We study the following problem: whether the compound system *S* can maintain “global order” in the situation of an increase of local disorder and if *S* can preserve the low entropy while other $S_{i}$ increase their entropies (may be essentially). We show that the entropy of a system as a whole can be constant, while the entropies of its parts rising. For classical systems, this is impossible, because the entropy of *S* cannot be less than the entropy of its subsystem $S_{i}$. And if a subsystems’s entropy increases, then a system’s entropy should also increase, by at least the same amount. However, within the quantum information theory, the answer is positive. The significant role is played by the entanglement of a subsystems’ states. In the absence of entanglement, the increasing of local disorder implies an increasing disorder in the compound system *S* (as in the classical regime). In this note, we proceed within a quantum-like approach to mathematical modeling of information processing by biosystems—respecting the quantum laws need not be based on genuine quantum physical processes in biosystems. Recently, such modeling found numerous applications in molecular biology, genetics, evolution theory, cognition, psychology and decision making. The quantum-like model of order stability can be applied not only in biology, but also in *social science and artificial intelligence*.

## 1. Introduction

This paper is motivated by Schrödinger’s book [1], in which he considered one of the most fundamental and intriguing problems of modern science: “What is life?” This was his attempt to proceed towards clarification of this problem on the basis of quantum physics and thermodynamics. Of course, from the purely biological viewpoint this attempt to resolve the basic problem of biology in the purely physical framework may be considered as very naive. Schrödinger by himself pointed out the casual nature of his approach. At the same the treatment of “What is life?” question in the purely physical framework can have its advantages; in particular, clarifying and cleaning the biological details may enlighten a few basic issues related to this question.

### 1.1. Order-Stability as a Distinguishing Feature of Biosystems

Schrödinger tried to find analogies between physical and biological systems’ functioning. And he emphasized *the amazing ability for order preservation as one of the basic features of functioning of biological systems*. He compared this feature with the thermodynamics of physical systems governed by the Second Law of Thermodynamics:

“What is the characteristic feature of life? When is a piece of matter said to be alive? It is alive when it goes on ‘doing something’, moving, exchanging material with its environment, and so forth, and that for a much longer period than we would expect of an inanimate piece of matter to ‘keep going’ under similar circumstances.”

Entropy is the basic measure of disorder in physics and information theory. To be stable, a biosystem should be able to control entropy and prevent its essential increase. (It is clear that entropy can fluctuate.) Schrödinger views the heuristic mechanism of entropy-stabilization through its emission from a system *S* to the environment E, that is, through an increase of disorder in E. Such a system’s behaviour does not match the laws of physics [1]: “When a system that is not alive is isolated or placed in a uniform environment, all motion usually comes to a standstill…” Schrödinger continues to speculate and suggests that *S* absorbs a flow of “negative entropy” from E. From the viewpoint of conventional physics, this notion is ambiguous and he points out that the mystery of life cannot be explained without the discovery of new physical laws.

### 1.2. Information Biology and Physics

The book [1] stimulated the creation of a new area of science which is nowadays known as information biology by emphasizing that order stability or even its improvement for the alive-state cannot be modelled solely in terms of the energy and matter flows between a biosystem *S* and the environment E. Biosystems should be viewed as *open systems* interacting with the physical and information components of the surrounding environment. Since the 1970s, information’s role was highlighted in biology, for example, the well known paper of Johnson [2] characterizing information theory as a “general calculus for biology”. As was pointed by Gatenby and Frieden [3], “it is clear that life without matter and energy is impossible, Johnson’s manuscript emphasizes that life without information is likewise impossible. Since the article, remarkable progress has been made towards understanding the informational fundament for life…” This information reconstruction of biology was closely related to the similar process in physics, starting with Wheeler’s *“It from bit”* [4] and to the recent *quantum information revolution*. The latter has led to an information reconsideration of quantum foundations [5,6,7,8,9,10,11]. Therefore, quantum information and open quantum systems [12] can contribute to the modelling of information interactions of the biosystem *S* and the environment E (see monograph [13]).

### 1.3. Quantum-Like Models

This is a good place to make the remark on genuine quantum and *quantum-like modeling* in biology. The first one is known as quantum biophysics and it describes genuine quantum processes in biosystems (see [14] for review). It operates on micro-scales, see, for example, the series of works [15,16,17,18,19,20,21,22] on modeling cognition from genuine quantum physical processes in the brain. Schrödinger’s book was the first step in this direction. (Maybe the global aim of quantum biophysics is too ambitious—to reduce biological functions, as say psychological functions, to quantum physical processes. The difference in scales, for space, time and temperature, is too big.).

In quantum-like modeling, a biosystem is characterized from the purely information processing viewpoint, that is, its size and other scales, say of temperature, are not important. As was shown in numerous studies (mainly in cognition, psychology and decision making, but even microbiology) [23,24,25,26,27,28,29,30,31,32,33,34,35,36,37,38,39,40,41,42,43,44,45,46,47,48,49,50,51,52,53,54,55,56,57,58,59,60,61,62,63,64,65,66,67,68,69,70], in some contexts biosystems process information in accordance with the quantum laws. Thus, they can be considered as quantum-like (although not genuinely quantum). In this paper, we proceed in the quantum-like framework. It covers even formal information processing in genuine quantum systems, that is, so to say software. Of course, quantum physical hardware differs crucially from hardware of macroscopic quantum-like information processors.

One may point to differences in the classical and quantum probabilistic descriptions [71,72,73] and the applicability of the quantum probability calculus and, hence, the quantum information theory to macroscopic biosystems. However, interrelations between these two descriptions is a complex problem of quantum foundations which we are not able to discuss in this paper (see, e.g., [74,75,76]). The main distinguishing feature of quantum(-like) information processing is operation with states’ superpositions. They represent unresolved uncertainty. This uncertainty is not reduced to classical probabilistic uncertainty, because incompatible variables cannot be realized on the same probability space. So, the same quantum state carries uncertainties for numerous incompatible variables. Such information processing saves a lot of computational resources. A biosystem (or social, or AI system, see Section 1.5), processes its state and then at each time it can select the concrete representation corresponding to some variable.

### 1.4. Order-Stability in a Biosystem Compounded of a Few Subsystems from a Quantum Information Approach

We model the order stability inside of a complex biosystem *S* that is composed of a few subsystems $S_{i},i=1,2\ldots ,N.$ We study the following problem:

*Can a compound system $S=(S_{i})$ preserve the “global order” in itself, in spite of the increase of local disorder—in its subsystems?*

In the mathematical framework, this question is formulated as follows:

*Can $S=(S_{i})$ preserve its entropy while some of its subsystems $S_{i}$ increase (may be essentially) their entropies?*

We show that within quantum information processing the answer is positive. However, within the classical information processing the increase of subsystem’s entropy automatically implies the increase of compound system’s entropy.

The key point is that in quantum theory the significant role is played by entanglement (cf. [67]), nonclassical correlations between the states of subsystems $S_{i}$ of S. In the absence of entanglement, entropy behaves classically.

We explore the following feature of quantum channels (dynamical maps describing the state evolution)—they can transfer non-entangled states into entangled. We present the scheme of the concrete quantum channels construction preserving the global entropy and increasing all local entropies. This feature of quantum channels is well known and widely used in quantum information theory and its applications to quantum computing. The novelty is in its exploring for modeling order-stability in biosystems.

We point to the model of entanglement production as an action realized by a special operator over given disentangled states which was proposed in articles [77,78]. This approach generalizes the standard quantum information scheme based on quantum channels and it is especially interesting for applications to biology, social science, management, and artificial intelligence. In such applications, one cannot exclude that information processing is based on more general quantum operators than quantum channels.

### 1.5. Other Applications: Social Science, Management, Artificial Intelligence, Information Retrieval

Our quantum-like framework and the result on entropy-stability can be applied not only to biosystems compounded of subsystems, say organs compounded of cells or organisms compounded of organs, or interacting neural networks in the brain, but also to social science and management (see the paper of Lawless [65] who also mentioned a coupling with Schrödinger’s book [1]), and artificial intelligence; in particular, to modeling behavior of future AI-systems which will be equipped with quantum processors—quantum computers, simulators, memory devices, internet based networks endowed with quantum cryptography. On the other hand, already nowadays the quantum-like ideology can stimulate design and development of Artificial Intelligence (AI)-systems equipped with classical processors, but exploring the quantum information processing. Some steps in this direction were done within the recent studies on quantum(-like) information retrieval (see, e.g., [79,80,81]): Algorithms based on the complex Hilbert state representation of information, but driven on classical computers, demonstrate superiority w.r.t. some parameters comparing with the traditional (“classical”) algorithms.

One of the distinguishing features of quantum-like modeling is that, for its output, the physical basis of information processors is not important. So, we can apply results of such modeling both to genuine quantum and macroscopic classical information processors and AI-systems.

For AI-systems, the main result of this paper is that systems equiped with quantum(-like) software are more stable than pure classical AI-systems. In classical AI, generation of disorder in any sybsystem $S_{i}$ has impact to the whole AI-system S. In quantum AI, entanglement between states of subsystems can prevent generation of global disorder from the local destabilization.

In social systems using quantum-like information processing, the global stability can be preserved, in spite of local destabilization. The crucial point is information processing in the form of superpositions, that is, without complete resolution of uncertainties.

### 1.6. The Problem of Self-Measurement

In Section 7, we briefly discuss the problem of self-measurements performed by biosystems within quantum measurement theory and more concretely the indirect measurement scheme [82]. Such self-measurements destroy entanglement and generate classical probabilistic mixtures.

## 2. Classical Entropy

### 2.1. Micro and Macrostates

Suppose that at some instant of time, a system *S* can be in one of states labeled by symbols $x_{j},j=1,2,\ldots ,n.$ We call them microstates of S; set $A_{S}=\left\{x_{j}\right\}.$ Let ρ be a probability distribution on $A_{S}$, that is, $\rho =(p\left(x\right):x\in A_{S}),$ where $p_{(}x)\geq 0,\sum_{x}p\left(x\right)=1.$ We call ρ a *macrostate, or simply state*.

For state ρ, entropy is defined as

(1) S(ρ)=−∑p(x)logp(x).

In bio applications (see Section 2.4), this quantity can be interpreted in the following way. Suppose that mcirostates of *S* can be “scanned” by other biosystems. Thus the microstate dynamics can be treated as signaling to other biosystems. For simplicity, we consider the discrete time dynamics; it generates the sequence of symbols

(2) $$X=x\left(\tau_{1}\right),\ldots ,x\left(\tau_{N}\right),\ldots ,$$

where τ is the time parameter of the microstate-dynamics. Mathematically this dynamics (signaling) is modeled as a random process. Under some conditions, the probability p(x) can be interpreted as the frequency probability - the limiting frequency of occurrence of the symbol *x* in the process’s trajectory X: $p\left(x\right)=\lim_{N\to \infty }N\left(x\right)/N,$ where N(x) is the number of occurrences of *x* in the sequence (2).

If system *S* is able to preserve its microstate, say one concrete $y\in A_{S},$ then p(y)=1 and p(x)=0,x≠y, and entropy S=0. (The microstate can fluctuate visiting *x*-states different from y, but not so often, as 0=limN(x)/N). In contrast, if the microstate of system *S* fluctuates covering uniformly $A_{S},$ then p(x)=1/n and entropy S=logn. Thus entropy can be used as the measure of state-stability, order preservation in S. The increase of entropy implies the decrease of information, the diminishing of order, and death, or at least decay. On the contrary, the decrease of entropy means the increase of information, the rise of order, and life or at least the improvement of self-organization.

### 2.2. Compound Systems

We are interested in compound systems $S=(S_{1},S_{2}).$ The (statistical) states of *S* are represented by probability distributions ρ=(p(x,y)), where $p_{x,y}\geq 0,\sum_{xy}p\left(x,y\right)=1.$ The entropy of *S* is given by

(3) $$S\left(\rho \right)=-\sum_{xy}p\left(x,y\right)\log p\left(x,y\right).$$

For a compound system, the states of its subsystems are given by the marginal probability distributions:

(4) $$\rho_{1}=(p_{1}\left(x\right)=\sum_{y}p\left(x,y\right),\;\rho_{2}=\left(p_{2}\left(y\right)=\sum_{x}p\left(x,y\right)\right),$$

and the corresponding entropies are

(5) $$S\left(\rho_{1}\right)=-\sum_{x}p_{1}\left(x\right)\log p_{1}\left(x\right),S\left(\rho_{2}\right)=-\sum_{j}p_{2}\left(y\right)\log p_{2}\left(y\right).$$

We can consider two bio-systems, say two cells, that communicate with each other: $S_{2}$ “feels” *x*-states of $S_{1}$ and vice verse: cell-signaling. Systems $S_{i}$ can represent as well neural networks in the brain, social systems, or AI-systems.

If $\rho =\rho_{1}⊗\rho_{2}$ (the direct product of probability measures), that is, probability $p\left(x,y\right)=p_{1}\left(x\right)p_{2}\left(y\right),$ then

(6) $$S\left(\rho \right)=S\left(\rho_{1}\right)+S\left(\rho_{2}\right).$$

Generally, additivity is violated and only the *subadditivity inequality* holds:

(7) $$S\left(\rho \right)\leq S\left(\rho_{1}\right)+S\left(\rho_{2}\right).$$

In the quantum case, the situation is the same. We now point out the specific classical constraint between the entropy of a compound system and subsystems’ entropies:

(8) $$S\left(\rho \right)\geq S\left(\rho_{i}\right).$$

Quantum information processing relaxes this constraint; in such processing the global order in a compound biosystem *S* can be preserved, in spite generating of local disorders in its subsystems $S_{i}.$

### 2.3. Stability of Global Order Is Possible Only with Stable Local Orders

Consider a model of signaling between biosystems based on recognition not of microstates, but macrostates. So, $S_{1}$ and $S_{2}$ communicate by recognition of the macrostates of each other (the probability distributions). There are two time scales, the fine time scale parameter τ and the rough time scale parameter *t* corresponding to the micro and macro state dynamics, respectively. The τ-scale dynamics determines macrostates evolving with the *t*-scale dynamics.

Suppose that state of *S* evolves in accordance with some dynamics t→ρ(t). It generates dynamics of subsystems’ states $t\to \rho_{i}\left(t\right).$ Suppose that initially *S* had very low entropy, $S(\rho_{0})=\epsilon <<1$ and suppose that it does not increase (at least essentially) with time, that is, S(ρ(t))<<1. Inequalities (8), (7) trivially imply inequality:

(9) $$S\left(\rho_{1}\left(t\right)\right)+S\left(\rho_{2}\left(t\right)\right)\leq 2S\left(\rho \left(t\right)\right).$$

Hence, stability of entropy S(ρ(t)) is possible only under assumption of stability of the entropy of each subsystem, $S(\rho_{i}\left(t\right)).$ In other words, preserving of global order is possible only under the condition of local orders preserving—in all subsystems.

### 2.4. Classical Information Processing in Biosystem

Now consider a complex biosystem containing a large ensemble of identical “elementary biological subsystems”, say cells; for example, an organism *S* with *N* cells; each of them can be in one of the microstates. Probability p(x) can be interpreted as the statistical (ensemble) probability $p\left(x\right)=\lim_{N\to \infty }N\left(x\right)/N,$ where N(x) is the number of cells in the state x. This leads to the statistical interpretation of entropy. (The ergodicity assumption gives the possibility of identification of the frequency probabilities with the statistical (ensemble) probabilities.).

Let system $S=(S_{1},S_{2})$ be a compound biosystem such that each $S_{j}$ contains a large number of “elementary biological subsystems”, say cells. Then, at each instance of time t, the macrostates of *S* and $S_{j};\rho \left(t\right),\rho_{j}\left(t\right),j=1,2,$ can be determined statistically. The previous considerations on their entropies are applicable even with the statistical interpretation of probabilities.

We have presented two different schemes for determination and recognitions of macrostates which are related to two different types of signaling, information exchange between biosystems, $S_{1}$ and $S_{2}:$

1. frequency (temporal),
2. statistical (ensemble).

In temporal framework, $S_{1}$ monitors the microstate of $S_{2}$ during some interval at the fine time scale τ and after each such time interval updates information about the macrostate of $S_{2}.$ At the rough time scale t, this update is treated as the instantaneous change of the macrostate; or in the language of infinitesimals—the state determination τ-interval Δt is infinitesimal w.r.t. to the rough scale and, $\rho_{i}\left(t+\Delta \right)$ is the result of the update of the state $\rho_{i}\left(t\right).$

In the ensemble framework, $S_{1}$ updates the microstate of $S_{2}$ via determination of intensity of realizations of the microstates of $S_{2}.$ Here the probability distribution p(x) can be interpreted as a field, the probability field. Of course, determination of this field of probability can neither be done instantaneously. The previous two scale scheme should be applied.

Generalization of these two interpretation-schemes to the quantum case is not straightforward.

## 3. Quantum Entropy

### 3.1. A Few Words about the Quantum Formalism

Denote by H a complex Hilbert space endowed with the scalar product 〈·|·〉. For simplicity, we assume that *it is finite dimensional*. The space of density operators is denoted by $S\left(H\right)$ The space of all linear operators in H is denoted by the symbol L(H). In turn, this is the complex Hilbert space with the scalar product, $\langle A|B\rangle =Tr\left(A^{☆}B\right).$ We shall also consider linear operators acting in L(H). They are called *superoperators*.

A pure quantum state is represented by a vector |ψ〉∈H that is normalized to 1, that is, 〈ψ|ψ〉=1. It can be represented as the density operator $\rho_{\psi }=|\psi \rangle \langle \psi |;$ this is the orthogonal projector on the vector |ψ〉. States which are not pure are called mixed.

### 3.2. Features of von Neumann Entropy

The von Neumann entropy is defined as

(10) S(ρ)=−Trρlnρ,

where ρ is a density operator.

There exists an orthonormal basis |j〉 consisting of eigenvectors of ρ, that is, $\rho |j\rangle =p_{j}|j\rangle$ (where $p_{j}\geq 0$ and $\sum_{j}p_{j}=1).$ In this basis, the matrix of the operator ρlnρ has the form $\mathrm{diag}(p_{j}\ln p_{j};)$ hence

(11) $$S\left(\rho \right)=-\sum_{j}p_{j}\ln p_{j}.$$

However, the von Neumann entropy has the classical form, but only w.r.t. this to special basis.

We present three basic properties of the von Neumann entropy.

1. S(ρ)=0 if and only if ρ is a pure quantum state, that is, ρ=|ψ〉〈ψ|.
2. For a unitary operator $U,S\left(U\rho U^{☆}\right)=S\left(\rho \right).$
3. The maximum of entropy is approached on the state $\rho_{\mathrm{disorder}}=I/N$ and $S(\rho_{\mathrm{disorder}})=\ln N,$ where *N* is the dimension of the state space.

It is natural to call $\rho_{\mathrm{disorder}}=I/N$ the state of maximal disorder.

### 3.3. Information Processing in Classical vs. Genuine Quantum and Quantum-Like Bio and AI Systems

Starting with Formula (11), one can try to proceed along the classical interpretational scheme. To consider vectors $x_{j}=|e_{j}\rangle$ as microstates a system S, and the density operator ρ as the macrostate of S: the operator form of representation of the probability distribution $p_{j}=p\left(x_{j}\right).$ However, another density operator has different basis and different set of microstates. One can try to overcome this problem by declaring the micro-state space as the unit sphere s(H) of Hilbert space H. Then each “macrostate” given by a density operator determines the probability distribution on the set of its basis states. One of the problems of such an approach is that, if some eigenvalues of ρ are degenerate, then the set of microstates is not not uniquely defined. As the most striking example, take the state ρ=I/N, where N=dimH. Then any basis in H can be considered as the set of its microstates and this operator ρ determines infinitely many different probability distributions on s(H). And some of them are mutually-singular, from measure-theoretic viewpoint. In fact, the situation is even more indefinite. For a density operator ρ, we can consider general decompositions of the form:

(12) $$\rho =-\sum_{j}q_{j}|\psi_{j}\rangle \langle \psi_{j}|,$$

where $q_{j}\geq 0,\sum_{j}q_{j}=1,$ and $\left(y_{j}=|\psi_{j}\rangle \right)$ is any set of pure states (pretending to be microstates). Then the probability distribution $q\left(y_{j}\right)=q_{j}$ on the subset $\left(y_{j}\right)$ of sphere s(H) can also be considered as a macrostate represented by operator ρ.

It seems that the classical picture of microstate–macrostate measure-theoretic interrelation is not applicable (at least straightforwardly) to the quantum case.

For the moment, we proceed with the formal mathematical model in which biosystems’ states are given by density operators. This approach matches perfectly the genuine quantum biophysics [14] and our study can be considered as justification of order stability in compound quantum biophysical systems, including stability of mental processing based on the “quantum brain model” in the spirit of papers [15,16,17,18,19,20,21,22]. The same can be said about the genuine quantum artificial intelligence based on quantum computers and simulators. However, our desire is to apply the order-stability result of this paper to quantum-like macroscopic biosystems. Some steps in this direction were done in works [63,83,84]. In the latter, the quantum-like information processing in the brain is generated via superposition representation of action potentials in neurons. This representation can be considered as qubit digitization of continuous action potentials and linear quantum(-like) dynamics as linearization of nonlinear classical electrochemical dynamics in neural networks.

In modeling order-stability in the quantum-like AI-devices, we can proceed with the formal mathematical representation of states by density operators which is realized on classical computing devices.

### 3.4. States of a Compound System and Its Subsystems, Entanglement

Let $S=(S_{1},S_{2})$ be a compound system represented in Hilbert space $H_{1}⊗H_{2}$ and let $\rho \in S\left(H_{1}⊗H_{2}\right).$ The states of its subsystems are calculated as the partial traces of ρ:

(13) $$\rho_{1}=Tr_{H_{2}}\;\rho ,\;\;\rho_{2}=Tr_{H_{1}}\;\rho ,$$

$\rho_{i}\in S\left(H_{i}\right).$

Consider now a pure state of *S* that is factorisable w.r.t. the tensor product structure, that is, $\left|\Psi \rangle =\right|\psi_{1}\rangle ⊗|\psi_{2}\rangle .$ States which are not represented in this form are called *entangled*. Entangled states play the crucial role in quantum information theory. In particular, this is the most important resource of quantum computations. They represent the correlations between subsystems of a quantum system. These correlations are nonclassical, in the sense that they cannot be adequately described by the classical probability, the Kolmogorov measure-theoretic axiomatics [71].

The definition of entanglement can be generalized to mixed states. A state $\rho \in S\left(H_{1}⊗H_{2}\right)$ is called separable if it can be represented in the form:

(14) $$\rho =\sum_{km}c_{km}\rho_{1}^{\left(k\right)}⊗\rho_{2}^{\left(m\right)},$$

where $\rho_{i}^{\left(j\right)}\in S\left(H_{i}\right),i=1,2.$ A compound state that cannot be represented in this form is called entangled. However, in this paper we shall consider only entanglement of pure states.

If the state ρ is factorisable, that is, $\rho =\rho_{1}⊗\rho_{2},$ then

(15) $$S\left(\rho \right)=S\left(\rho_{1}\right)+S\left(\rho_{2}\right),$$

cf. (6). Generally, as in the classical case (see (7)), we have only subadditivity

(16) $$S\left(\rho \right)\leq S\left(\rho_{1}\right)+S\left(\rho_{2}\right).$$

However, in contrast to the classical case, it can happen that

(17) $$S\left(\rho \right)<S\left(\rho_{i}\right),$$

cf. (8). We shall explore this distinguishing feature of the quantum information measure of disorder.

Consider now the pure state $\rho_{\Psi }=|\Psi \rangle \langle \Psi |,$ where $|\Psi \rangle \in H_{1}⊗H_{2}.$ The states of the systems $S_{i}$ are pure if and only if |Ψ〉 is separable. Thus, for an entangled state $\rho_{\Psi },$ the states $\rho_{i}$ are always mixed states.

This fact is important for our further study. It implies that, for an entangled pure state, the entropies of subsystem’s states $S(\rho_{i})>0,$ because $\rho_{i}$ is not pure.

### 3.5. Compound Systems; Quantum Channels

Consider evolution of the state of the compound system $S=\left(S_{1},S_{2}\right),\;\rho \left(t\right)=\Lambda_{t}\rho_{0}$ and the corresponding evolution of the states of $S_{i},$

(18) $$\rho_{1}\left(t\right)=Tr_{H_{2}}\Lambda_{t}\rho_{0},\;\rho_{2}\left(t\right)=Tr_{H_{1}}\Lambda_{t}\rho_{0}.$$

In the framework of open quantum systems theory, for each t, a state’s evolution of *S* is represented by *a quantum channel*—trace-preserving completely positive map (superoperator) acting in the space $L\left(H_{1}⊗H_{2}\right).$

Each subsystem $S_{i}$ of the compund system *S* can be considered as an open quantum system. In the case of the isolated system S, system $S_{2}$ plays the role of the environment of system $S_{1}$ and vice verse. If *S* is not isolated, the environment of $S_{1}$ includes $S_{2}$ and the environment of $E_{S}$ of S.

## 4. Stability of Global Order, in Spite of the Increase of Local Disorder

We are interested in the condition of order-stability in the compound system *S* in the situation of disorder-increasing in its subsystems $S_{i}.$ Suppose that initially all entropies were very small $S\left(\rho_{0}\right),S\left(\rho_{0i}\right)<<1.$ Suppose now that subsystems’ entropies started to increase and in the process of evolution they can increase essentially. Can $S(\rho_{0})$ preserve its value (or increase only slightly)?

In this note, we consider the case of factorizable pure initial state of the compound system S, that is, $\rho_{0}=\rho_{01}⊗\rho_{02}.$

The simplest model of such behavior is based on the unitary evolution of S, that is, one parameteric group of unitary operators $U_{t}:H_{1}⊗H_{2}\to H_{1}⊗H_{2}.$ In this case, $\Lambda_{t}\rho_{0}=U_{t}\rho_{0}U_{t}^{☆}.$ Such dynamics transfer pure states into pure states. Hence, $S\left(\Lambda_{t}\rho_{0}\right)=S\left(\rho_{0}\right)=0.$ If $\rho_{0}$ corresponds to a separable pure state, then $S(\rho_{0i})=0,i=1,2,$ as well.

If the quantum channel $\Lambda_{t}$ transfers a separable state into an entangled state, then $\rho_{i}\left(t\right),i=1,2,$ are mixed states and, hence, they have positive entropy. Thus, our desire is to construct *a unitary evolution operator that can transfer separable states into entangled states*. It is well-known that such operators exist and they are widely used in quantum computations. For readers convenience (and especially by taking into account that this issue is directed to experts in cognition, psychology, and decision making and not in quantum information theory), we present the well known examples of such operators for state spaces of an arbitrary (finite) dimension. These operators are explicitly expressed through orthonormal bases in Hilbert spaces $H_{i}$ and an entropy increase can be calculated explicitly.

## 5. Complex Systems

A biosystem *S* is typically composed of a large number of subsystems $S_{i},i=1,2,\ldots ,M$ (say genes, proteins, cells, organs, neural networks). Let subsystem $S_{i}$ be represented in Hilbert space $H_{i}.$ The compound system *S* is represented in the tensor product $H={⊗}_{i=1}H_{i}.$ For quantum state $\rho \in S\left(H\right),$ states of the subsystems are given by partial traces

$$\rho_{j}=Tr_{{⊗}_{i\neq j}H_{i}}\rho .$$

Let $\Lambda_{t}$ be a quantum channel describing the dynamics of the compound state, $\rho \left(t\right)=\Lambda_{t}\rho_{0};$ then the states of subsystems evolves as

$$\rho_{j}\left(t\right)=Tr_{{⊗}_{i\neq j}H_{i}}\Lambda_{t}\rho_{0}.$$

For the fixed subsystem $S_{j},$ the system $S_{j}^{\prime }={\left(S_{i}\right)}_{i\neq j}$ plays the role of its environment (in the case of isolated S). We are interested in generalization of condition (17) for i=1,2,…,M. However, even in the case M=2 considered in this paper calculations are long. We do not want to overshadow the main idea of compound-stability by even longer calculations. Although calculations for an arbitrary *M* are more complicated, but it is clear that desired quantum channels can be constructed, especially for spaces of the dimension $\dim\;H=2^{M},$ for *M* qubit spaces.

## 6. Quantum Channel Preserving Compound System’s Entropy, in Spite of Increasing of Its Subsystems’ Entropies

The constructions of the desired quantum channel for subsystem’s state spaces of dimensions N=2 and N>2 are different. In the latter case, the expressions for the von Neumann entropies of the subsystems $S_{i},i=1,2,$ contain the factor log(N−2). Therefore, we consider these cases separately.

### 6.1. Two Subsystems with Qubit State Spaces

Let $H_{1}$ and $H_{2}$ be $C^{2}$ and $\left\{|x_{0}^{\left(i\right)}\rangle ,|x_{1}^{\left(i\right)}\rangle \right\}$ be orthonormal bases in $H_{i}$ $\left(i=1,2\right)$. We define a completely positive channel Λ from $S\left(H_{1}⊗H_{2}\right)$ to $S\left(H_{1}⊗H_{2}\right)$ by

$$\Lambda \left(•\right)\equiv V\left(•\right)V,$$

where *V* is a linear map from $H_{1}⊗H_{2}$ to $H_{1}⊗H_{2}$ given by

$$\begin{array}{ccc} V & \equiv & \sqrt{\frac{1}{2}}\left(|x_{0}^{\left(1\right)}\rangle ⊗|x_{0}^{\left(2\right)}\rangle +|x_{1}^{\left(1\right)}\rangle ⊗|x_{1}^{\left(2\right)}\rangle \right)\langle x_{0}^{\left(1\right)}|⊗\langle x_{0}^{\left(2\right)}|\\ & & +\sqrt{\frac{1}{2}}\left(|x_{0}^{\left(1\right)}\rangle ⊗|x_{0}^{\left(2\right)}\rangle -|x_{1}^{\left(1\right)}\rangle ⊗|x_{1}^{\left(2\right)}\rangle \right)\langle x_{1}^{\left(1\right)}|⊗\langle x_{1}^{\left(2\right)}|\\ & & +\sqrt{\frac{1}{2}}\left(|x_{0}^{\left(1\right)}\rangle ⊗|x_{1}^{\left(2\right)}\rangle +|x_{1}^{\left(1\right)}\rangle ⊗|x_{0}^{\left(2\right)}\rangle \right)\langle x_{0}^{\left(1\right)}|⊗\langle x_{1}^{\left(2\right)}|\\ & & +\sqrt{\frac{1}{2}}\left(|x_{0}^{\left(1\right)}\rangle ⊗|x_{1}^{\left(2\right)}\rangle -|x_{1}^{\left(1\right)}\rangle ⊗|x_{0}^{\left(2\right)}\rangle \right)\langle x_{1}^{\left(1\right)}|⊗\langle x_{0}^{\left(2\right)}|. \end{array}$$

We remark that the operator *V* is unitary (see, e.g., [85]). Thus this channel is noiseless—it is given by the unitary dynamics.

Let $\rho_{0}$ be an initial compound state on $H_{1}⊗H_{2}$ of the form:

$$\rho_{0}=\left(|x_{0}^{\left(1\right)}\rangle ⊗|x_{0}^{\left(2\right)}\rangle \right)\left(\langle x_{0}^{\left(1\right)}|⊗\langle x_{0}^{\left(2\right)}|\right).$$

We remark that this is the density operator corresponding to a *pure state* and the von Neumann entropy of $\rho_{0}$ equals zero:

$$S\left(\rho_{0}\right)=0.$$

We point out that the pure state under consideration is separable (non-entangled) and hence the two marginal states of $\rho_{0}$ are given by density operators corresponding to pure states:

$$\rho_{01}=|x_{0}^{\left(1\right)}\rangle \langle x_{0}^{\left(1\right)}|,\;\rho_{02}=|x_{0}^{\left(2\right)}\rangle \langle x_{0}^{\left(2\right)}|.$$

The von Neumann entropy of two marginal states $\rho_{01}$ and $\rho_{02}$ are equal to zero:

$$S\left(\rho_{01}\right)=0,\;S\left(\rho_{02}\right)=0.$$

The final compound state $\Lambda \left(\rho_{0}\right)$ transmitted through the CP channel is

$$\Lambda \left(\rho_{0}\right)=\sqrt{\frac{1}{2}}\left(|x_{0}^{\left(1\right)}\rangle ⊗|x_{0}^{\left(2\right)}\rangle +|x_{1}^{\left(1\right)}\rangle ⊗|x_{1}^{\left(2\right)}\rangle \right)\sqrt{\frac{1}{2}}\left(\langle x_{0}^{\left(1\right)}|⊗\langle x_{0}^{\left(2\right)}|+\langle x_{1}^{\left(1\right)}|⊗\langle x_{1}^{\left(2\right)}|\right).$$

We emphasize that this is the density operator corresponding to an entangled pure state.

The entropy of transformed state $\Lambda \left(\rho_{0}\right)$ coincides with the entropy of the initial state:

$$S\left(\Lambda \left(\rho_{0}\right)\right)=0=S\left(\rho_{0}\right).$$

The two marginal states of $\Lambda \left(\rho_{0}\right)$ are

$$\Lambda_{1}\rho_{01}=\frac{1}{2}|x_{0}^{\left(1\right)}\rangle \langle x_{0}^{\left(1\right)}|+\frac{1}{2}|x_{1}^{\left(1\right)}\rangle \langle x_{1}^{\left(1\right)}|,\;\Lambda_{2}\rho_{02}=\frac{1}{2}|x_{0}^{\left(2\right)}\rangle \langle x_{0}^{\left(2\right)}|+\frac{1}{2}|x_{1}^{\left(2\right)}\rangle \langle x_{1}^{\left(2\right)}|.$$

The von Neumann entropy of two marginal states $\Lambda_{1}\rho_{01}$ and $\Lambda_{2}\rho_{02}$ are

$$\begin{array}{ccc} S\left(\Lambda_{i}\rho_{0i}\right) & = & \log2>S\left(\rho_{0i}\right)=S\left(\Lambda \left(\rho_{0}\right)\right)=0. \end{array}$$

Thus the entropies of both subsystems increased for log2-amount, but the entropy of *S* preserves its zero value.

### 6.2. Two Subsystems with *N*-dimensional State Spaces

We expand the above setting to N×N compound systems (N≥3).

Let $H_{1}$ and $H_{2}$ be $C^{N}$ and ${\left\{|x_{k}^{\left(i\right)}\rangle \right\}}_{k=0}^{N-1}$ be orthonormal bases in $H_{i}$ $\left(i=1,2\right)$. We define a completely positive channel Λ from $S\left(H_{1}⊗H_{2}\right)$ to $S\left(H_{1}⊗H_{2}\right)$ by

$$\Lambda \left(•\right)\equiv V\left(•\right)V,$$

where *V* is a linear map from $H_{1}⊗H_{2}$ to $H_{1}⊗H_{2}$ given by

$$V=\sum_{k,\ell =0}^{N-1}|\varphi_{k,\ell }\rangle \langle x_{k}^{\left(1\right)}|⊗\langle x_{\ell }^{\left(2\right)}|,$$

where

$$\begin{array}{ccc} |\varphi_{k,\ell }\rangle & = & \frac{2}{N}\sum_{j=0}^{N-1}\alpha_{k,\ell ,j}|x_{j}^{\left(1\right)}\rangle ⊗|x_{j+k\;\mathrm{mod}\;N}^{\left(2\right)}\rangle \\ \alpha_{k,\ell ,j} & = & \left\{\begin{array}{cc} -\frac{N-2}{2} & \left(j=\ell \right)\\ 1 & \left(j\neq \ell \right) \end{array}\right.\;\left(k=0,1,2,\cdots ,N-1\right) \end{array}$$

$$\begin{array}{ccc} V & = & \frac{2}{N}\left(\left(-\frac{N-2}{2}\right)|x_{0}^{\left(1\right)}\rangle ⊗|x_{0}^{\left(2\right)}\rangle +|x_{1}^{\left(1\right)}\rangle ⊗|x_{1}^{\left(2\right)}\rangle +\cdots +|x_{N-1}^{\left(1\right)}\rangle ⊗|x_{N-1}^{\left(2\right)}\rangle \right)\langle x_{0}^{\left(1\right)}|⊗\langle x_{0}^{\left(2\right)}|\\ & & +\frac{2}{N}\left(|x_{0}^{\left(1\right)}\rangle ⊗|x_{0}^{\left(2\right)}\rangle +\left(-\frac{N-2}{2}\right)|x_{1}^{\left(1\right)}\rangle ⊗|x_{1}^{\left(2\right)}\rangle +\cdots +|x_{N-1}^{\left(1\right)}\rangle ⊗|x_{N-1}^{\left(2\right)}\rangle \right)\langle x_{0}^{\left(1\right)}|⊗\langle x_{1}^{\left(2\right)}|\\ & & +\cdots +\\ & & +\frac{2}{N}\left(|x_{0}^{\left(1\right)}\rangle ⊗|x_{0}^{\left(2\right)}\rangle +|x_{1}^{\left(1\right)}\rangle ⊗|x_{1}^{\left(2\right)}\rangle +\cdots +\left(-\frac{N-2}{2}\right)|x_{N-1}^{\left(1\right)}\rangle ⊗|x_{N-1}^{\left(2\right)}\rangle \right)\langle x_{0}^{\left(1\right)}|⊗\langle x_{N-1}^{\left(2\right)}|\\ & & +\frac{2}{N}\left(\left(-\frac{N-2}{2}\right)|x_{0}^{\left(1\right)}\rangle ⊗|x_{1}^{\left(2\right)}\rangle +|x_{1}^{\left(1\right)}\rangle ⊗|x_{2}^{\left(2\right)}\rangle +\cdots +|x_{N-1}^{\left(1\right)}\rangle ⊗|x_{0}^{\left(2\right)}\rangle \right)\langle x_{1}^{\left(1\right)}|⊗\langle x_{0}^{\left(2\right)}|\\ & & +\frac{2}{N}\left(|x_{0}^{\left(1\right)}\rangle ⊗|x_{1}^{\left(2\right)}\rangle +\left(-\frac{N-2}{2}\right)|x_{1}^{\left(1\right)}\rangle ⊗|x_{2}^{\left(2\right)}\rangle +\cdots +|x_{N-1}^{\left(1\right)}\rangle ⊗|x_{0}^{\left(2\right)}\rangle \right)\langle x_{1}^{\left(1\right)}|⊗\langle x_{1}^{\left(2\right)}|\\ & & +\cdots +\\ & & +\frac{2}{N}\left(|x_{0}^{\left(1\right)}\rangle ⊗|x_{1}^{\left(2\right)}\rangle +|x_{1}^{\left(1\right)}\rangle ⊗|x_{2}^{\left(2\right)}\rangle +\cdots +\left(-\frac{N-2}{2}\right)|x_{N-1}^{\left(1\right)}\rangle ⊗|x_{0}^{\left(2\right)}\rangle \right)\langle x_{1}^{\left(1\right)}|⊗\langle x_{N-1}^{\left(2\right)}|\\ & & +\frac{2}{N}\left(\left(-\frac{N-2}{2}\right)|x_{0}^{\left(1\right)}\rangle ⊗|x_{2}^{\left(2\right)}\rangle +|x_{1}^{\left(1\right)}\rangle ⊗|x_{3}^{\left(2\right)}\rangle +\cdots +|x_{N-1}^{\left(1\right)}\rangle ⊗|x_{1}^{\left(2\right)}\rangle \right)\langle x_{2}^{\left(1\right)}|⊗\langle x_{0}^{\left(2\right)}|\\ & & +\frac{2}{N}\left(|x_{0}^{\left(1\right)}\rangle ⊗|x_{2}^{\left(2\right)}\rangle +\left(-\frac{N-2}{2}\right)|x_{1}^{\left(1\right)}\rangle ⊗|x_{3}^{\left(2\right)}\rangle +\cdots +|x_{N-1}^{\left(1\right)}\rangle ⊗|x_{1}^{\left(2\right)}\rangle \right)\langle x_{2}^{\left(1\right)}|⊗\langle x_{1}^{\left(2\right)}|\\ & & +\cdots +\\ & & +\frac{2}{N}\left(|x_{0}^{\left(1\right)}\rangle ⊗|x_{2}^{\left(2\right)}\rangle +|x_{1}^{\left(1\right)}\rangle ⊗|x_{3}^{\left(2\right)}\rangle +\cdots +\left(-\frac{N-2}{2}\right)|x_{N-1}^{\left(1\right)}\rangle ⊗|x_{1}^{\left(2\right)}\rangle \right)\langle x_{2}^{\left(1\right)}|⊗\langle x_{N-1}^{\left(2\right)}|\\ & & +\cdots \cdots \cdots +\\ & & +\frac{2}{N}\left(\left(-\frac{N-2}{2}\right)|x_{0}^{\left(1\right)}\rangle ⊗|x_{N-1}^{\left(2\right)}\rangle +|x_{1}^{\left(1\right)}\rangle ⊗|x_{0}^{\left(2\right)}\rangle +\cdots +|x_{N-1}^{\left(1\right)}\rangle ⊗|x_{N-2}^{\left(2\right)}\rangle \right)\langle x_{N-1}^{\left(1\right)}|⊗\langle x_{0}^{\left(2\right)}|\\ & & +\frac{2}{N}\left(|x_{0}^{\left(1\right)}\rangle ⊗|x_{N-1}^{\left(2\right)}\rangle +\left(-\frac{N-2}{2}\right)|x_{1}^{\left(1\right)}\rangle ⊗|x_{0}^{\left(2\right)}\rangle +\cdots +|x_{N-1}^{\left(1\right)}\rangle ⊗|x_{N-2}^{\left(2\right)}\rangle \right)\langle x_{N-1}^{\left(1\right)}|⊗\langle x_{1}^{\left(2\right)}|\\ & & +\cdots +\\ & & +\frac{2}{N}\left(|x_{0}^{\left(1\right)}\rangle ⊗|x_{N-1}^{\left(2\right)}\rangle +|x_{1}^{\left(1\right)}\rangle ⊗|x_{0}^{\left(2\right)}\rangle +\cdots +\left(-\frac{N-2}{2}\right)|x_{N-1}^{\left(1\right)}\rangle ⊗|x_{N-2}^{\left(2\right)}\rangle \right)\langle x_{N-1}^{\left(1\right)}|⊗\langle x_{N-1}^{\left(2\right)}|. \end{array}$$

The operator *V* is unitarity (see, e.g., [85]). Hence, this channel is noiseless.

Let $\rho_{0}$ be an initial compound state on $H_{1}⊗H_{2}$ denoted by

$$\rho_{0}=\left(|x_{0}^{\left(1\right)}\rangle ⊗|x_{0}^{\left(2\right)}\rangle \right)\left(\langle x_{0}^{\left(1\right)}|⊗\langle x_{0}^{\left(2\right)}|\right).$$

One finds the von Neumann entropy of $\rho_{0}$ such that

$$S\left(\rho_{0}\right)=0.$$

The two marginal states of $\rho_{0}$ are

$$\rho_{01}=|x_{0}^{\left(1\right)}\rangle \langle x_{0}^{\left(1\right)}|,\;\rho_{02}=|x_{0}^{\left(2\right)}\rangle \langle x_{0}^{\left(2\right)}|.$$

The von Neumann entropy of the two marginal states $\rho_{01}$ and $\rho_{02}$ are

$$S\left(\rho_{01}\right)=0,\;S\left(\rho_{02}\right)=0.$$

The final compound state $\Lambda \left(\rho_{0}\right)$ transmitted through the CP channel is

$$\begin{array}{ccc} \Lambda \left(\rho_{0}\right) & = & \frac{2}{N}\left(\left(-\frac{N-2}{2}\right)|x_{0}^{\left(1\right)}\rangle ⊗|x_{0}^{\left(2\right)}\rangle +\sum_{k=1}^{N-1}|x_{k}^{\left(1\right)}\rangle ⊗|x_{k}^{\left(2\right)}\rangle \right)\\ & & \frac{2}{N}\left(\left(-\frac{N-2}{2}\right)\langle x_{0}^{\left(1\right)}|⊗\langle x_{0}^{\left(2\right)}|+\sum_{k=1}^{N-1}\langle x_{k}^{\left(1\right)}|⊗\langle x_{k}^{\left(2\right)}|\right). \end{array}$$

We also have the von Neumann entropy of $\Lambda \left(\rho_{0}\right)$ is

$$S\left(\Lambda \left(\rho_{0}\right)\right)=0=S\left(\rho_{0}\right)$$

The two marginal states of $\Lambda \left(\rho_{0}\right)$ are

$$\begin{array}{ccc} \Lambda_{1}\rho_{01} & = & \frac{{\left(N-2\right)}^{2}}{N^{2}}|x_{0}^{\left(1\right)}\rangle \langle x_{0}^{\left(1\right)}|+\frac{4}{N^{2}}\sum_{k=1}^{N-1}|x_{k}^{\left(1\right)}\rangle \langle x_{k}^{\left(1\right)}|,\;\\ \Lambda_{2}\rho_{02} & = & \frac{{\left(N-2\right)}^{2}}{N^{2}}|x_{0}^{\left(2\right)}\rangle \langle x_{0}^{\left(2\right)}|+\frac{4}{N^{2}}\sum_{k=1}^{N-1}|x_{k}^{\left(2\right)}\rangle \langle x_{k}^{\left(2\right).}| \end{array}$$

The von Neumann entropy of the two marginal states $\Lambda_{1}\rho_{01}$ and $\Lambda_{2}\rho_{02}$ are

$$\begin{array}{ccc} S\left(\Lambda_{i}\rho_{0i}\right) & = & 2\log N-\frac{2{\left(N-1\right)}^{2}}{N^{2}}\log\left(N-2\right)-\frac{8\left(N-1\right)}{N^{2}}\log2>S\left(\rho_{0i}\right)=S\left(\Lambda \left(\rho_{0}\right)\right)=0. \end{array}$$

Consider the above general formulas for the case N=3. Let $\rho_{0}$ be an initial compound state on $H_{1}⊗H_{2}$ denoted by

$$\rho_{0}=\left(|x_{0}^{\left(1\right)}\rangle ⊗|x_{0}^{\left(2\right)}\rangle \right)\left(\langle x_{0}^{\left(1\right)}|⊗\langle x_{0}^{\left(2\right)}|\right)$$

One finds the von Neumann entropy of $\rho_{0}$ such that

$$S\left(\rho_{0}\right)=0.$$

The two marginal states of $\rho_{0}$ are

$$\rho_{01}=|x_{0}^{\left(1\right)}\rangle \langle x_{0}^{\left(1\right)}|,\;\rho_{02}=|x_{0}^{\left(2\right)}\rangle \langle x_{0}^{\left(2\right).}|$$

The von Neumann entropy of the two marginal states $\rho_{01}$ and $\rho_{02}$ are

$$S\left(\rho_{01}\right)=0,\;S\left(\rho_{02}\right)=0.$$

The final compound state $\Lambda \left(\rho_{0}\right)$ transmitted through the CP channel is

$$\begin{array}{ccc} \Lambda \left(\rho_{0}\right) & = & \frac{2}{3}\left(|x_{0}^{\left(1\right)}\rangle ⊗|x_{0}^{\left(2\right)}\rangle +|x_{1}^{\left(1\right)}\rangle ⊗|x_{1}^{\left(2\right)}\rangle -\frac{1}{2}|x_{2}^{\left(1\right)}\rangle ⊗|x_{2}^{\left(2\right)}\rangle \right)\\ & & \frac{2}{3}\left(\langle x_{0}^{\left(1\right)}|⊗\langle x_{0}^{\left(2\right)}|+\langle x_{1}^{\left(1\right)}|⊗\langle x_{1}^{\left(2\right)}|-\frac{1}{2}\langle x_{2}^{\left(1\right)}|⊗\langle x_{2}^{\left(2\right)}|\right). \end{array}$$

We also have the von Neumann entropy of $\Lambda \left(\rho_{0}\right)$ is

$$S\left(\Lambda \left(\rho_{0}\right)\right)=0=S\left(\rho_{0}\right)$$

The two marginal states of $\Lambda \left(\rho_{0}\right)$ are

$$\begin{array}{ccc} \Lambda_{1}\rho_{01} & = & \frac{4}{9}|x_{0}^{\left(1\right)}\rangle \langle x_{0}^{\left(1\right)}|+\frac{4}{9}|x_{1}^{\left(1\right)}\rangle \langle x_{1}^{\left(1\right)}|+\frac{1}{9}|x_{2}^{\left(1\right)}\rangle \langle x_{2}^{\left(1\right)}|,\;\\ \Lambda_{2}\rho_{02} & = & \frac{4}{9}|x_{0}^{\left(2\right)}\rangle \langle x_{0}^{\left(2\right)}|+\frac{4}{9}|x_{1}^{\left(2\right)}\rangle \langle x_{1}^{\left(2\right)}|+\frac{1}{9}|x_{2}^{\left(2\right)}\rangle \langle x_{2}^{\left(2\right).}| \end{array}$$

The von Neumann entropy of two marginal states $\Lambda_{1}\rho_{1}$ and $\Lambda_{2}\rho_{02}$ are

$$\begin{array}{ccc} S\left(\Lambda_{i}\rho_{0i}\right) & = & 2\log3-\frac{16}{9}\log2>S\left(\rho_{0i}\right)=S\left(\Lambda \left(\rho_{0}\right)\right)=0. \end{array}$$

## 7. Quantum Measurement Theory: Self-Observations in Biosystems

Up to this section, our presentation was done without even mentioning the cornerstone of quantum mechanics—quantum measurement theory. In the latter, the crucial role is played by interaction between a system γ and a measurement apparatus M. By the Copenhagen interpretation outputs of quantum measurements are not properties of a system, but outputs of the complex process of γ−M interaction. Properties of a system are inapproachable directly; they are reflected in outputs of the pointer of M. It is important to separate system γ from measurement apparatus M. This separation is the delicate point of quantum measurement theory. And the situation is even more airy for self-observations performed by biosystems. Who observes whom?

We suggest resolving the issue of self-observations via straightforward application of the methodology of quantum mechanics. Subsystems of a biosystem *S* (at least some of them) can perform measurements on other subsystems. In the simplest case, $S=(S_{1},S_{2})$ and say subsystem $S_{2}$ performs observation on subsystem $S_{1},$ that is, $S_{2}$ plays the role of a measurement apparatus.

The most adequate description of such processes can be given within the indirect measurement scheme going back to von Neumann [86] (see also Ozawa [82] for modern formalization and coupling with theory of quantum instruments).

## 8. The Indirect Measurement Scheme

The indirect measurement scheme can be represented as the block of following interrelated components:

- the states of the systems γ and the apparatus M; they are represented in complex Hilbert spaces H and K, respectively;
- the unitary operator *U* representing the interaction-dynamics for the compound system Γ=(γ,M);
- the meter observable $M_{A}$ giving outputs of the pointer of the apparatus M.

It is assumed that the compound system Γ is isolated. The dynamics of pure states of the compound system is described by the Schrödinger equation:

(19) $$i\frac{d}{dt}|\Psi \rangle \left(t\right)=H|\Psi \rangle \left(t\right),\;|\Psi \rangle \left(0\right)={|\Psi \rangle }_{0},$$

where *H* is it Hamiltonian of Γ and $|\Psi \rangle \left(t\right)={U\left(t\right)|\Psi \rangle }_{0};$ U(t) is the unitary operator $U\left(t\right)=e^{-itH}.$ And Hamiltonian *H* has the form: $H_{\Gamma }=H_{\gamma }⊗I+I⊗H_{M}+H_{\gamma ,M},$ where $H_{\gamma }:H\to H,H_{M}:K\to K$ are Hamiltonians of γ and M, respectively, and $H_{\gamma ,M}\in H⊗K\to H⊗K$ is Hamiltonian of interaction between systems γ and M.

Suppose that we want to measure an observable on the system γ, which is represented by Hermitian operator A, acting in system’s state space H. The *indirect measurement model* for measurement of the *A*-observable was introduced by Ozawa in [82] as a “(general) measuring process”; this is a quadruple

$$(K,\sigma ,U,M_{A}),$$

consisting of a Hilbert space K, a density operator $\sigma \in S\left(K\right),$ a unitary operator *U* on the tensor product of the state spaces of γ and M, U:H⊗K→H⊗K, and a Hermitian operator $M_{A}$ on K.

Here, K represents the states of the apparatus *M*, *U* describes the time-evolution of system Γ, σ describes the initial state of the apparatus *M* before the start of measurement, and the Hermitian operator $M_{A}$ is the meter observable of the apparatus *M* (say the pointer of M). This operator represents indirectly outcomes of an observable *A* for the system γ.

The probability distribution $\mathrm{Pr}\{A=x∥\rho_{\gamma }\}$ in the system state $\rho_{\gamma }\in S\left(H\right)$ is given by

(20) $$\mathrm{Pr}\left\{A=x∥\rho_{\gamma }\right\}=Tr\left[\left(I⊗E^{M_{A}}\left(x\right)\right)U\left(\rho_{\gamma }⊗\sigma \right)U^{☆}\right],$$

where $E^{M_{A}}\left(x\right)$ is the spectral projection of $M_{A}$ for the eigenvalue x. We reall that operator $M_{A}$ is Hermitian. In the finite dimensional case, it can be represented in the form:

(21) $$M_{A}=\sum_{k}x_{k}E^{M_{A}}\left(x_{k}\right),$$

where $\left(x_{k}\right)$ is the set of its eigenvalues and $E^{M_{A}}\left(x_{k}\right)$ is the projector on the subspace of eigenvectors corresponding to eigenvalue $x_{k}.$

The change of the state $\rho_{\gamma }$ of the system γ caused by the measurement for the outcome A=x is represented with the aid of the map $I_{A}\left(x\right)$ in the space of density operators defined as

(22) $$I_{A}\left(x\right)\rho_{\gamma }=Tr_{K}\left[\left(I⊗E^{M_{A}}\left(x\right)\right)U\left(\rho_{\gamma }⊗\sigma \right)U^{☆}\right],$$

where $Tr_{K}$ is the partial trace over K. We remark that the map $x\mapsto I_{A}\left(x\right)$ is a *quantum instrument* [82] (see [69] for simple and brief introduction to theory of quantum instrument theory).

### 8.1. Biosystems

Consider now a biosystem *S* that is compound of two subsystems $S_{1}$ and $S_{2}.$ In the above measurement scheme, we set $\gamma =S_{1},M=S_{2},$ and $H=H_{1},K=H_{2},\rho_{\gamma }=\rho_{01},\sigma =\rho_{02},$ the unitary operator *U* determines the quantum channel Λ. If the initial density operators correspond to pure states, then, as we have seen, the unitary evolution of the state of *S* can generate the density operator corresponding to an entangled pure state expressing the special character of correlations between the states of subsystems $S_{1}$ and $S_{2}.$ However, quantum instrument $I_{A}\left(x\right)$ generates a mixed state, that is, measurement on subsystem $S_{1}$ perfromed by subsystem $S_{2}$ destroys special quantum-information correlations inside S. The state of *S* is given by

(23) $$\rho_{x}=I⊗E^{M_{A}}\left(x\right)U\left(\rho_{\gamma }⊗\sigma \right)U^{☆}/Tr\;I⊗E^{M_{A}}\left(x\right)U\left(\rho_{\gamma }⊗\sigma \right)U^{.}☆$$

This is the state resulting from self-observation in S.

Generally, *S* can be composed of a large number *m* of subsystems $S_{i}.$ They all can perform observations on each other. Each such observation interrupts the unitary evolution of S. As model examples, we can consider cell-signaling and processing of information in the brain.

### 8.2. Consciousness

In the brain, there is an information system which is specialized on brain’s self-observations. We can call it *consciousness*, denote it by the symbol C. (We repeat that systems under consideration are information systems. So, *C* need not be identified with the special spatial area of the brain. It can spatially distributed over different areas of the brain.) It is a subsystem of the “information brain”, that is, compound system *S* of all information processors in the physical brain; *C* contains numerous measurement apparatuses which specialized on observations on the states of various subsystems $S_{i}$ of S.

## 9. Concluding Discussion

Here we present a new approach to the problem of order-stability in biosystems formulated by Schrödinger in 1944 [1]. This approach is based on the quantum-like paradigm realized in the framework of the open quantum systems theory. The following particular problem is studied: *preservation of order-stability by a biosystem S as a compound of subsystems performing some biological functions generating disorder-increasing*. In the modelling, we explored the features of quantum information processing, especially the constancy of an isolated quantum information system entropy and the possibility to generate entangled states. The quantum-like model is purely informational, that is, biosystems are considered as information processors; for each subsystem $S_{i},$ the rest of the compound system *S* is treated as the *information environment*. The order-stability has the meaning of stability of information processing in S. Thus, this paper is a part of the information approach to physics and biology, from Wheeler’s *“It from bit”* [4] to the recent *information interpretation* of quantum theory [5,6,7,8,9,10,11] and Johnson’s emphasize that life without information processing is impossible [2]. Once again, we stress that this approach is not rigidly coupled to the micro-world, but it supports strongly *the quantum-like paradigm - context sensitive systems, for example, biosystems can process information in accordance with the laws of quantum information theory*.

In this paper, we considered the simplest situation of an isolated compound biosystem S. The next step is modeling order stability of the quantum information state of a compound open system *S* interacting with the information environment $E_{S}.$ Its state dynamics is non-unitary. In such a model, disorder in the biosystem *S* is coming both from outside, namely from the information environment $E_{S},$ and from inside, that is to say, the subsystems $S_{i}$ of S.

The phenomenon of life is not reduced to order stability. However, even consistent modelling of information exchange stability in a complex biosystem is a step towards clarification of this phenomenon. The authors hope that this paper matches with Schrödinger vision [1] of information processes in biosystems (within a modern quantum information representation).

We also discussed the problem of *self-observations in biosystems* within the indirect measurement scheme of quantum observations. This is the complex problem and in this paper we restricted our considerations to the brief discussion. We plan to turn to this problem in one of further publications.

The result of this paper on order stability in the whole system, while order decreases in its subsystems, is also applicable to social and AI-systems processing information in accordance with the quantum theory.

We can distinguish two types of AI-systems:

- Systems equipped with genuine quantum information processing devices, say quantum computers or simulators.
- Systems equipped with classical information processing devices, say classical digital or analog computers, realizing quantum(-like) information processing.

Personally I do not share the generally high expectation for successful realization of genuine quantum physical computing project, especially hopes that such quantum devices can be useful for AI-systems, say robots. I think that quantum information processing based on classical computational devices has better perspectives. But, since in science it is always difficult to make prognoses for future development, both types of AI-systems, genuine quantum and quantum-like, have to be studied. In future, the output of this paper may become useful for modeling behavior of collectives composed of quantum and quantum-like robots and other AI-systems.

However, the main impact of this paper is in clarification of order stability in biosystems as a consequence of quantum(-like) information processing. We hope that this is a step (of course, a little step) towards clarification of phenomenon of life.

## Data Availability

Data sharing not applicable.
